# Historical, New, and Reemerging Links between Human and Animal Health

**DOI:** 10.3201/eid1012.041037

**Published:** 2004-12

**Authors:** Nina Marano, Marguerite Pappiaoanou

**Affiliations:** * Centers for Disease Control and Prevention, Atlanta, Georgia, USA

**Keywords:** animal health, emerging disease, introduction, zoonoses, agriculture, wildlife

**Figure 1:**
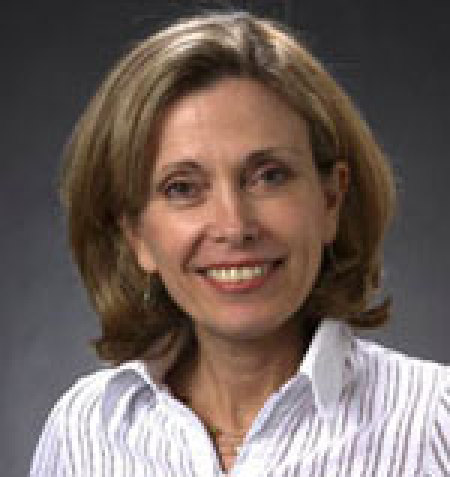
Dr. Marano is the associate director for veterinary medicine and public health within the National Center for Infectious Diseases at CDC. She is responsible for promoting partnerships between the animal health and public health sectors. She works closely with the American Veterinary Medical Association and the Association of American Veterinary Medical Colleges to integrate veterinary specialists into research to improve detection, prevention, management, and control of emerging zoonotic diseases.

**Figure 2:**
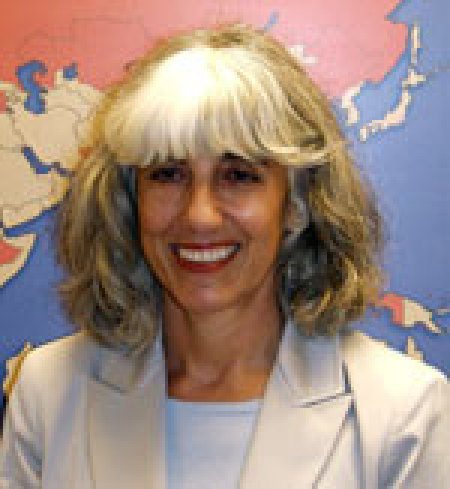
Dr. Pappaioanou is the associate director for science and policy within the Office of Global Health at the Centers for Disease Control and Prevention (CDC). Her areas of interest are to bring the public health and animal health sectors together and to study the impact of increasing wildlife and human interaction on emerging infectious diseases.

Introduction by guest editors Nina Marano (Figure 1) and Marguerite Pappiaoanou (Figure 2).

A wide spectrum of microbes and infectious diseases have been transmitted from domesticated and wild animals to humans for thousands of years (*1*). In the last 5 years, infectious diseases such as West Nile virus infection and monkeypox have appeared in North America, and severe acute respiratory syndrome and avian influenza have emerged on a global scale. We learn from each new event, and we hope that we will be sufficiently prepared to prevent, or to detect and effectively respond to, the next event. These diseases, which disregard national borders, include new infections caused by changes or evolution of existing organisms (e.g., recent report of rabies virus transmission through organ transplantation) (*2*), known infections expanding to new geographic locations (e.g., emergence of West Nile virus in North America beginning with the United States in 1999), previously unrecognized infections appearing in areas undergoing ecologic transformation (e.g., Nipah virus in humans and swine in Malaysia) (*3*), new infections reemerging as a result of antimicrobial resistance developing in existing agents (e.g., emergence of infections caused by multidrug-resistant strains of *Salmonella* Newport) (*4*), or breakdowns in public health measures (e.g., *Mycobacterium bovis* tuberculosis [5]) (*6*).

The World Health Organization has defined zoonoses as those diseases and infections naturally transmitted between nonhuman vertebrate animals and humans (*7*), and emerging zoonotic disease as a "zoonosis that is newly recognized or newly evolved or that has occurred previously but shows an increase in incidence or expansion in geographical, host or vector range" (*8*). Strikingly, 75% of emerging infectious diseases have been identified as zoonotic in origin (*9*).

All of the following factors have been identified as risk factors for the emergence of zoonotic diseases: international travel; global trade; increasing interactions among humans, wildlife, and exotic and domesticated food and companion animals; human behavior; rapid microbial adaptation; changing climates and ecosystems; and changing livestock management methods (*10*). Gaining a better understanding of zoonotic disease emergence, prevention, and control requires quality basic and applied research, which results from extensive interaction and collaboration among professionals from multiple disciplines. These disciplines should include ecology; entomology; occupational medicine; pathology; animal and human behavioral science; epidemiology; biostatistics; economics; clinical veterinary and human medicine; human and veterinary public health; environmental health; and regulatory, wildlife, and agricultural sciences.

Emerging Infectious Diseases was established to promote the recognition of new and reemerging infectious diseases around the world and to improve the understanding of factors involved in disease emergence, prevention, and elimination. It is appropriate, therefore, that an entire issue of this journal be devoted to the topic of emerging and reemerging zoonotic diseases. This issue features articles from multiple countries that encompass a wide range of diseases and disease agents, including tularemia, Nipah virus, prion diseases, West Nile virus, cryptosporidiosis, hantavirus, bartonellosis, salmonellosis, parastrongylus, and lyssavirus. Multiple species are involved in transmission (e.g., wildlife, companion animals, fish, and amphibians) and a myriad of human behavioral risk factors (e.g., pet ownership, contact between pets and wildlife, direct contact with farm animals or wildlife) for these diseases.

The artwork featured on the cover of this December issue emphasizes the theme of humans living in harmony with animals. We hope this theme issue promotes greater awareness among our readers of the strong link between human and animal health and underscores the importance of establishing new partnerships between human and animal health, agricultural, natural resource, environmental, and other sectors to truly achieve a "Peaceable Kingdom."
